# Virulence, pathology, and pathogenesis of Pteropine orthoreovirus (PRV) in BALB/c mice: Development of an animal infection model for PRV

**DOI:** 10.1371/journal.pntd.0006076

**Published:** 2017-12-14

**Authors:** Kazutaka Egawa, Masayuki Shimojima, Satoshi Taniguchi, Noriyo Nagata, Hideki Tani, Tomoki Yoshikawa, Takeshi Kurosu, Shumpei Watanabe, Shuetsu Fukushi, Masayuki Saijo

**Affiliations:** 1 United Graduate School of Veterinary Sciences, Gifu University, Gifu, Japan; 2 Department of Virology I, National Institute of Infectious Diseases, Tokyo, Japan; 3 Department of Pathology, National Institute of Infectious Diseases, Tokyo, Japan; 4 Department of Virology, University of Toyama, Toyama, Japan; NIAID Integrated Research Facility, UNITED STATES

## Abstract

**Background:**

Cases of acute respiratory tract infection caused by Pteropine orthoreovirus (PRV) of the genus *Orthoreovirus* (family: *Reoviridae*) have been reported in Southeast Asia, where it was isolated from humans and bats. It is possible that PRV-associated respiratory infections might be prevalent in Southeast Asia. The clinical course of PRV is not fully elucidated.

**Methods:**

The virulence, pathology, and pathogenesis of two PRV strains, a human-borne PRV strain (isolated from a patient, who returned to Japan from Bali, Indonesia in 2007) and a bat-borne PRV (isolated from a bat [*Eonycteris spelaea*] in the Philippines in 2013) were investigated in BALB/c mice using virological, pathological, and immunological study methods.

**Results:**

The intranasal inoculation of BALB/c mice with human-borne PRV caused respiratory infection. In addition, all mice with immunity induced by pre-inoculation with a non-lethal dose of PRV were completely protected against lethal PRV infection. Mice treated with antiserum with neutralizing antibody activity after inoculation with a lethal dose of PRV showed a reduced fatality rate. In this mouse model, bat-borne PRV caused respiratory infection similar to human-borne PRV. PRV caused lethal respiratory disease in an animal model of PRV infection, in which BALB/c mice were used.

**Conclusions:**

The BALB/c mouse model might help to accelerate research on the virulence of PRV and be useful for evaluating the efficacy of therapeutic agents and vaccines for the treatment and prevention of PRV infection. PRV was shown for the first time to be a causative virus of respiratory disease on the basis of Koch’s postulations by the additional demonstration that PRV caused respiratory disease in mice through their intranasal inoculation with PRV.

## Author summary

It is assumed that Pteropine orthoreovirus (PRV) is a causative agent of acute respiratory tract infection (RTI) in humans. PRV was isolated from patients and fruit bats in Southeast Asia. Furthermore, the genome of PRV was detected in patients with respiratory symptoms, suggesting that PRV causes RTI in humans. There is a potential for PRV to cause RTIs in tropical or sub-tropical regions in Southeast Asia more widely than thought. The virulence, pathology, and pathogenesis of PRV in BALB/c mice were elucidated in detail. To develop specific countermeasures and prophylactics against PRV infection, an animal model of lethal PRV infection is needed, and we developed such a model using BALB/c mice. This model allows investigation of the pathogenicity of PRV and evaluation of the efficacy of drugs and vaccines and might help to accelerate research on the virology of PRV.

## Introduction

Pteropine orthoreovirus (PRV), a member of genus *Orthoreovirus* in the family *Reoviridae*, was originally isolated from the heart blood of a grey-headed flying fox (*Pteropus poliocephalus*) in Australia in 1968 [1].

PRV was isolated from a patient with respiratory tract infection (RTI) as a causative agent in Malaysia in 2006 [2]. Seven patients with symptoms of influenza-like illness, such as fever, cough, and sore throat, caused by PRV were reported between 2006 and 2017 [2–8]. Three patients with PRV infection were reported in Malaysia in 2006 and 2010 [2, 3, 5, 8]. Four cases of PRV infection imported from Indonesia to Japan and Hong Kong were identified in 2007, 2009, and 2010 [4, 6–8]. The presence of these RTI cases in Southeast Asia suggests that PRV might be the causative viral pathogen of RTI. Some patients with PRV also showed the symptoms of abdominal pain, watery diarrhea, and vomiting [3–5, 8]. Antibodies to PRV were detected in 13% of the residents of Tioman Island, Malaysia [2], and 4.4% of patients with nonspecific symptoms in central Vietnam [9]. Furthermore, PRV genomes were detected in 17% of patients with RTIs in Negeri Sembilan state, Malaysia [10]. These reports raise the concern that the prevalence of human PRV infection in Southeast Asia might be higher than previously thought. However, the disease spectrum and the pathogenesis of PRV infection in humans also remain unclear.

Fourteen strains of PRV have been isolated from fruit bats (*Pteropus poliocephalus*, *P*. *hypomelanus*, *P*. *vampyrus*, *Rousettus leschenaultia*, *Eonycteris spelaea*, and *R*. *amplexicaudatus*) in Australia, Malaysia, Indonesia, PR China, and the Philippines from 1968 to date [1, 8, 11–15]. The Indonesia/2010 strain was isolated from the salivary swab of *P*. *vampyrus* imported from Indonesia to Italy in 2010 [14]. PRV-neutralizing antibodies were also detected in 83% of fruit bat species (*R*. *amplexicaudatus*, *E*. *spelaea*, and *Macroglossus minimus*) in the Philippines, suggesting that PRV is generally prevalent in some species of wild bats in Southeast Asia [15]. It is still not known whether PRV causes illnesses in fruit bats [8], whereas bat-borne PRV is a potentially pathogenic to humans. Therefore, it is important to characterize both human-borne and bat-borne PRV.

A PRV strain isolated from a patient with RTI was found to be lethal in C3H mice, but the virulence and pathology of this strain in mice were not investigated in detail [16]. In the present study, the virulence, pathology, and pathogenesis of PRV in BALB/c mice were elucidated to validate respiratory disease caused by PRV and to develop an animal model of PRV infection.

## Materials and methods

### Viruses and cells

Two PRV strains that were isolated in previous studies [7, 15] were used in this study. The PRV strain Miyazaki-Bali/2007 (PRV-MB) was isolated from a patient with PRV infection, who returned to Japan from Bali, Indonesia in 2007 [7, 17]. The PRV strain Samal-24 (PRV-Samal-24) was isolated from *E*. *spelaea* in the Philippines in 2013 [15]. The nucleotide sequences of the 10 segments of each of these two PRV strains are deposited in GenBank (Table 1).

**Table 1:** GenBank accession numbers for the nucleotide sequences of the 10 RNA genome segments of the PRV-MB and PRV-Samal-24 strains used in this study.

Strain	Virus genome segment									
	S1	S2	S3	S4	M1	M2	M3	L1	L2	L3
PRV-MB	AB908284	AB908285	AB908286	AB908287	AB908281	AB908282	AB908283	AB908278	AB908279	AB908280
PRV-Samal-24	LC110198	LC110199	LC110200	LC110201	LC110202	LC110203	LC110204	LC110205	LC110206	LC110207

PRVs were propagated in human embryonic kidney-derived 293FT cells (Thermo Fisher Scientific, Inc.) for the preparation of the working virus solution. Cells infected with each strain of PRV were cultured at 37°C in Dulbecco’s modified eagle’s medium (DMEM; Sigma-Aldrich Co., LLC) supplemented with 5% heat-inactivated fetal bovine serum (FBS) and 1% antibiotics (penicillin and streptomycin; Pen-Strep, Thermo Fisher Scientific, Inc.) (DMEM-5FBS). After 2 days of culture, the medium was centrifuged at 800 × *g* for 5 min to remove cellular debris. The supernatant was overlaid onto 20% sucrose in a 50 ml tube (Becton Dickinson, Ltd.) and centrifuged at 100,000 × *g* for 2 h to concentrate the virus. The concentrated viruses were dissolved with DMEM with 2% FBS and 1% Pen-Strep (DMEM-2FBS), and the aliquots were stored at -80°C until use.

### Determination of infectious dose of PRV with a plaque assay

The infectious dose of each virus was determined in a plaque assay in Vero cell (ATCC, CCL-81) monolayers as described previously [7]. The cells were inoculated with a serially diluted virus solution of PRV-MB or PRV-Samal-24 and incubated for 1 h at 37°C for adsorption. The cell monolayers were washed with phosphate buffered saline solution (PBS), and the cells were cultured with DMEM-5FBS supplemented with 0.8% agarose for 2 days at 37°C. Plaque was visualized by staining the cells with neutral red solution. Plaques were counted, and the virus titers were calculated in plaque-forming units per milliliter (PFU/ml).

### Mice

Nine-week-old female BALB/c mice (Japan SLC, Inc.) were used. The mice used were healthy and weighed approximately 20 g.

### Determination of 50% lethal dose for PRV-MB and PRV-Samal-24

The mice, which were anesthetized with a combination of ketamine (100 mg/kg) and xylazine (4 mg/kg) in 0.9% sodium chloride solution, were inoculated with each strain of PRV. Five mice per group were intranasally inoculated with 1.0 × 10^3^ to 1.0 × 10^6^ PFU of each PRV strain in 20 μl DMEM-2FBS. The clinical signs and body weight of the mice were monitored for 14 days, and the 50% lethal dose (LD_50_) of PRV (for mice) was calculated according to the method of Reed and Muench [18]. Mice that were intranasally inoculated with 20 μl DMEM-2FBS (vehicle) were used as the control. The changes in body weight and the survival rates were plotted using the GraphPad Prism software program (GraphPad Software, Inc.) and were analyzed statistically by a one-way ANOVA.

### Quantitative detection of the PRV genome in organs and blood

Five mice were intranasally inoculated with 1.0 × 10^5^ PFU of the PRV-MB or PRV-Samal-24 strain as described above. The mice were sacrificed on the 5th or 6th day post-infection (DPI), and then blood and the organs (the head including the brain and nasal cavity, trachea, lung, liver, kidney, spleen, and intestine) were collected. The viral RNA load in each organ and blood was determined by a quantitative real-time RT-PCR (qRT-PCR) as described below.

### Determination of viral RNA load with a quantitative real-time RT-PCR

Blood samples were collected from the mice (5 per group) infected with each strain of PRV by cardiac puncture after euthanasia. Each of the blood samples was mixed with Isogen LS (Wako Pure Chemical Industries, Ltd.), and total RNA was extracted from each blood sample according to the manufacturer’s instructions. The organs and tissues; the brain, nasal cavity, trachea, lung, heart, liver, spleen, kidney, and intestine were collected. These samples were immediately submerged in RNAlater (Ambion, Life Technologies, Inc.) and stored at -80°C until use. Total RNA was extracted using Isogen (Wako Pure Chemical Industries, Ltd.) according to the manufacturer’s instructions. The viral copy numbers were determined with a qRT-PCR as follows. The forward and reverse primers and probe were specifically designed according to the nucleotide sequence of the outer-capsid protein (OCP) region in the S4 segment of PRV-MB or to that of PRV-Samal-24. The sequences of the forward primer, the reverse primer, and the probe for the amplification of the PRV-MB genome were 5’-CATTGTCACTCCGATCATGG-3’, 5’-TGGGAGTGTGCAGAGCATAG-3’ (Eurofins Genomics, Inc.), and FAM/5’-GTAGGTATGCCACTCGTGGAATCC-3’/TAMRA (Sigma-Aldrich Co. LLC.), respectively. The sequences of the forward primer, the reverse primer, and the probe for the amplification of the PRV-Samal-24 genome were 5’-CAATTTCCACTCGTTCGTTG-3’, 5’- GATGGTGTGGAAACGGATAC -3’ (Eurofins Genomics, Inc.), and FAM/5’- GACCAGACCAGATACGTGGAATCC -3’/TAMRA (Sigma-Aldrich Co. LLC.), respectively. The qRT-PCRs were performed using a Light Cycler 96 system (Roche Diagnostics, Ltd.) with a QuantiTect Probe RT-PCR Kit (Qiagen, Ltd.). The Light Cycler experimental protocol was as follows: reverse transcription (50°C for 30 min), denaturation (95°C for 15 min), and 45 cycles of amplification and quantification (94°C for 15 s and 60°C for 60 s), followed by a final cooling step at 40°C for 30 s. In this study, the standard controls for PRV-MB and PRV-Samal-24 were 10-fold serial dilutions of the plasmid DNA containing the S4 segments of PRV-MB and PRV-Samal-24, respectively. The viral copy numbers in the samples were calculated as the ratio of the copy numbers of each standard control. The viral copy numbers were plotted using the GraphPad Prism software program, and the results were statistically analyzed by a one-way ANOVA. The viral RNA detection limits in the blood, trachea, and other tissues were determined to be 2.5 × 10^3^ copies/ml, 1.6 × 10^3^–5.0 × 10^3^ copies/0.1 g, and 2.5 × 10^3^ copies/0.1 g, respectively. One PFU was equivalent to 2.9 copies of viral RNA.

### Quantification of the infectious PRV in the organs

Two mice were intranasally inoculated with 1.0 × 10^6^ PFU of the PRV-MB strain or of PRV-Samal-24 strain as described above. The mice were sacrificed on the 4th DPI, and organs (the head including the brain and the nasal cavity, trachea, lung, liver, kidney, and intestine) of the mice were collected. The infectious virus titer in each organ was determined with a plaque assay as described below. Each organ collected was immediately submerged in DMEM-2FBS, homogenized and centrifuged at 800 × *g* for 5 min to remove debris. The supernatant fraction was collected and stored at -80°C until use. The virus titer in the supernatant fraction was determined in a plaque assay in Vero cell monolayers as described previously [7]. The virus titers were plotted using the GraphPad Prism software program. The virus titer detection limit was determined to be 2.4 × 10^1^ PFU/0.1 g.

### Time-course analysis in the lung of mice infected with PRV

The mice intranasally infected with 1.0 × 10^3^ PFU of PRV-MB (PRV-MB-1.0×10^3^ PFU mice) and those intranasally infected with 1.0 × 10^5^ PFU of PRV-MB (PRV-MB-1.0×10^5^ PFU mice) were sacrificed by exposure to excess isoflurane, and the lungs were collected on the 1st, 3rd, and 5th DPI (5 mice per group each day). The viral RNA loads in the lungs were determined with qRT-PCR. Pathological analyses of the lungs were performed by immunohistochemical (IHC) analysis as described below.

### Histopathology and immunohistochemistry

The collected tissues were stained with hematoxylin and eosin (H&E) for histopathology. An IHC analysis was performed for the detection of PRV antigen in the tissues. The IHC analysis methods were the same as those described previously except for the antigen detection antibody [19]. The sections were deparaffinized by placing them in a retrieval solution (pH 6) (Nichirei Biosciences, Inc.), followed by heat-treatment with an autoclave at 121°C for 10 min. The polyclonal antibody to the OCP (S4 segment) of PRV-MB raised in a rabbit by immunization with the antigen (OCP antibody) was used for the IHC detection of PRV antigen [20]. The OCP antibody used in the IHC analysis reacted specifically with OCP antigens of PRV [20]. To validate whether the OCP antibody reacts with the mouse lungs non-specifically, the lung tissues of 6-month-old BALB/c mice infected with severe acute respiratory syndrome coronavirus (SARS-mouse-lung), in which severe inflammation was shown, and those of the mice inoculated with mock solution (mock-mouse-lung) were tested by IHC analysis [21]. The samples showed a negative reaction in the IHC analysis (S1 Fig), indicating that the OCP antibody does not react non-specifically with the mouse lungs with inflammation and that the positive signals detected in the IHC analysis indicate the presence of the OCP of PRV. As the negative control, normal rabbit serum (NRS; Dako, Ltd.) was used in IHC analysis. After treatment, the sections were reacted with the OCP antibody or NRS and then washed with PBS. The sections were incubated with Nichirei-Histofine Simple Stain Mouse MAX PO (R) (Nichirei Biosciences, Inc.) according to the manufacturer’s instructions. The peroxidase activity was detected with 3, 3’-diaminobenzidine (Sigma-Aldrich Co. LLC.), and the sections were counterstained with hematoxylin.

### Induction of immunity in mice by infection with PRV followed by lethal PRV infection

Five mice were intranasally inoculated with either DMEM-2FBS containing 1.0 × 10^3^ PFU (non-lethal dose) of PRV-MB or DMEM-2FBS (control). Serum was separated from the blood collected through the caudal vein on the 27th DPI by centrifugation. The serum was tested for the PRV-MB neutralizing antibody titers as described previously [9, 15]. In addition, mice that were pre-inoculated with PRV-MB or control were re-inoculated with 1.0 × 10^5^ PFU (lethal dose) of PRV-MB on the 35th day after the first inoculation with PRV-MB or control. The clinical signs and body weight were monitored for 14 days. The mice that showed >25% initial body weight loss were euthanized. Their body weight changes and survival rates were plotted using the GraphPad Prism software program.

### Treatment of mice with antiserum after PRV lethal infection

Twenty-five mice were intranasally inoculated with 1.0 × 10^3^ PFU (non-lethal dose) of PRV-MB followed by a second intranasal inoculation with 1.0 × 10^5^ PFU of PRV-MB 3 weeks after the first infection. The mice were then intranasally inoculated once more with 1.0 × 10^5^ PFU of PRV-MB 3 weeks after the second infection. On the 5th day after the third inoculation, the mice were sacrificed and blood was collected by cardiac puncture. Serum was separated by centrifugation. Mouse serum, which was collected from the 25 control mice without inoculation with PRV-MB, was used as the control serum.

The serum was diluted 4-fold with PBS. Five mice per group were intranasally infected with 1.0 × 10^5^ PFU of PRV-MB, and then the diluent of the serum (100 μL) was administered once daily until the mice showed >25% initial body weight loss for a maximum of 5 days. Serum was administered at just after 1 h after inoculation, or on the 1st, 2nd, 3rd, and 4th DPI. The diluent of the control serum (100 μL) was used for mock treatment. The mice that showed >25% initial body weight loss were euthanized. The body weight changes and survival rates were plotted using the GraphPad Prism software program.

### Ethics statement

The animal studies were carried out in strict accordance with the Guidelines for Proper Conduct of Animal Experiments of the Science Council of Japan and in strict compliance with animal husbandry and welfare regulations. All animal experiments were approved by the Committee on Experimental Animals at the National Institute of Infectious Diseases (NIID) in Japan (Approval Nos. 215016, 116086, and 116082). All of the animals infected with PRV were handled in biosafety level 3 animal facilities, in accordance with the guidelines of the NIID. The mice were inoculated with virus solution under proper anesthesia, and all efforts were made to minimize any potential pain and distress. After inoculation, the animals were monitored once a day during the study period. A humane endpoint was introduced for all mice with >25% initial body weight loss.

## Results

### Symptoms and viral loads in BALB/c mice infected with PRV-MB

**Fig 1:**
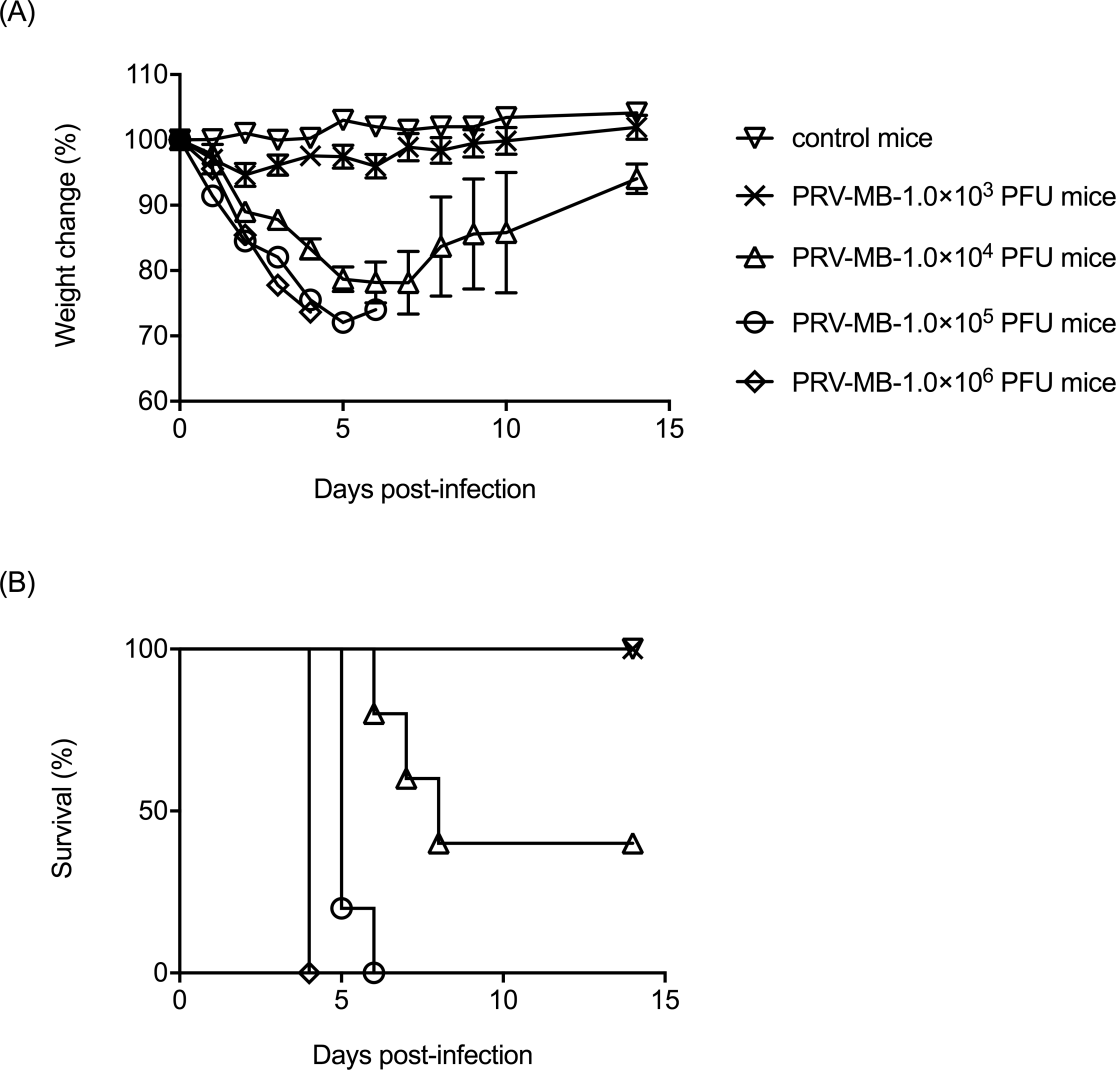
**Changes in the body weight (A) and survival rate (B) of BALB/c mice intranasally inoculated with 1.0 × 10**^**3**^**–1.0 × 10**^**6**^ **PFU of PRV-MB and the control mice.** The mice infected with each dose of PRV-MB are shown (5 mice per group). Error bars in A indicate the standard error.

The PRV-MB-1.0×10^5^ PFU mice or the PRV-MB-1.0×10^6^ PFU mice developed symptoms (piloerection, slowness in movement, anorexia, and weight loss) from the 2nd DPI. All of the mice died by the 6th DPI (Fig 1). The severity of the symptoms in the PRV-MB-1.0×10^4^ PFU mice was less than that in the PRV-MB-1.0×10^5^ PFU mice or the PRV-MB-1.0×10^6^ PFU mice, and 3 of the 5 mice died by the 8th DPI. The extent of body weight loss in the PRV-MB-1.0×10^3^ PFU mice was greater than that in the control mice. The PRV-MB-1.0×10^3^ PFU mice did not show any symptoms other than body weight loss. The LD_50_ of PRV-MB in the BALB/c mice was determined to be 6.8 × 10^3^ PFU/head.

**Fig 2:**
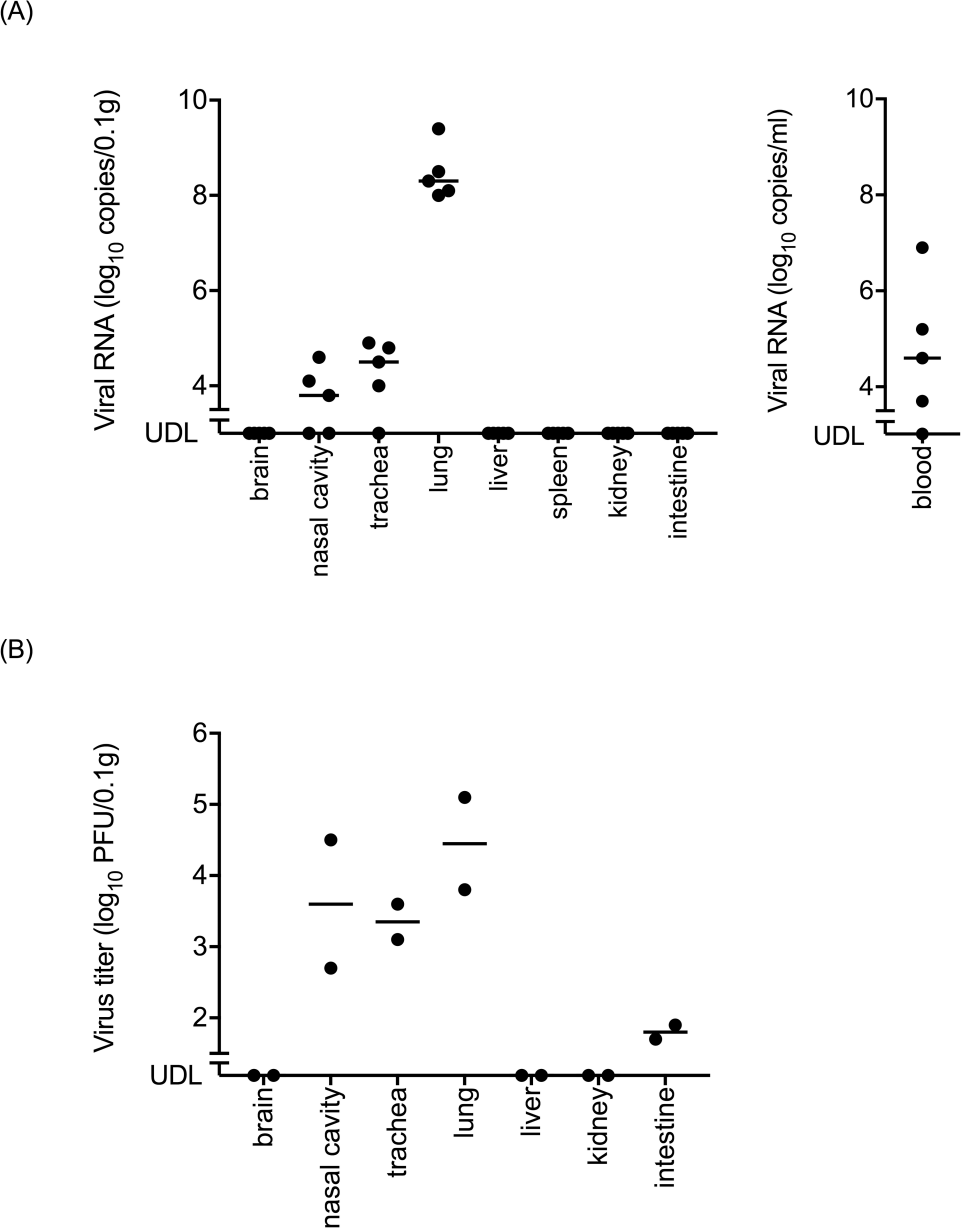
**Viral RNA loads in each organ and the blood (A) and infectious virus titers in each organ (B) of BALB/c mice infected with PRV-MB.** (A) The organs and blood samples were obtained from the PRV-MB-1.0×10^5^ PFU mice on the 5th or 6th DPI (5 mice per group). The left and right panels indicate the viral RNA loads in the organs and the blood of the mice, respectively. (B) The organs were obtained from the PRV-MB-1.0×10^6^ PFU mice on the 4th DPI (2 mice per group). UDL, under detection limit.

The level of viral RNA in the lungs (average level, 6.9 × 10^8^ copies/0.1 g) was higher than those in the other organs (Fig 2A, left panel). Viral RNA was detected in the blood (maximum level of 7.5 × 10^6^ copies/ml) (Fig 2A, right panel). In contrast, viral RNA was not detected in the brain, heart, liver, spleen, kidney, and intestine. The infectious virus was detected mainly in respiratory organs (Fig 2B). The titer in the lungs (average virus titer, 6.4 × 10^4^ PFU/0.1 g) was the highest among the organs tested.

### Pathology of the lower respiratory tract in BALB/c mice infected with PRV-MB

**Fig 3:**
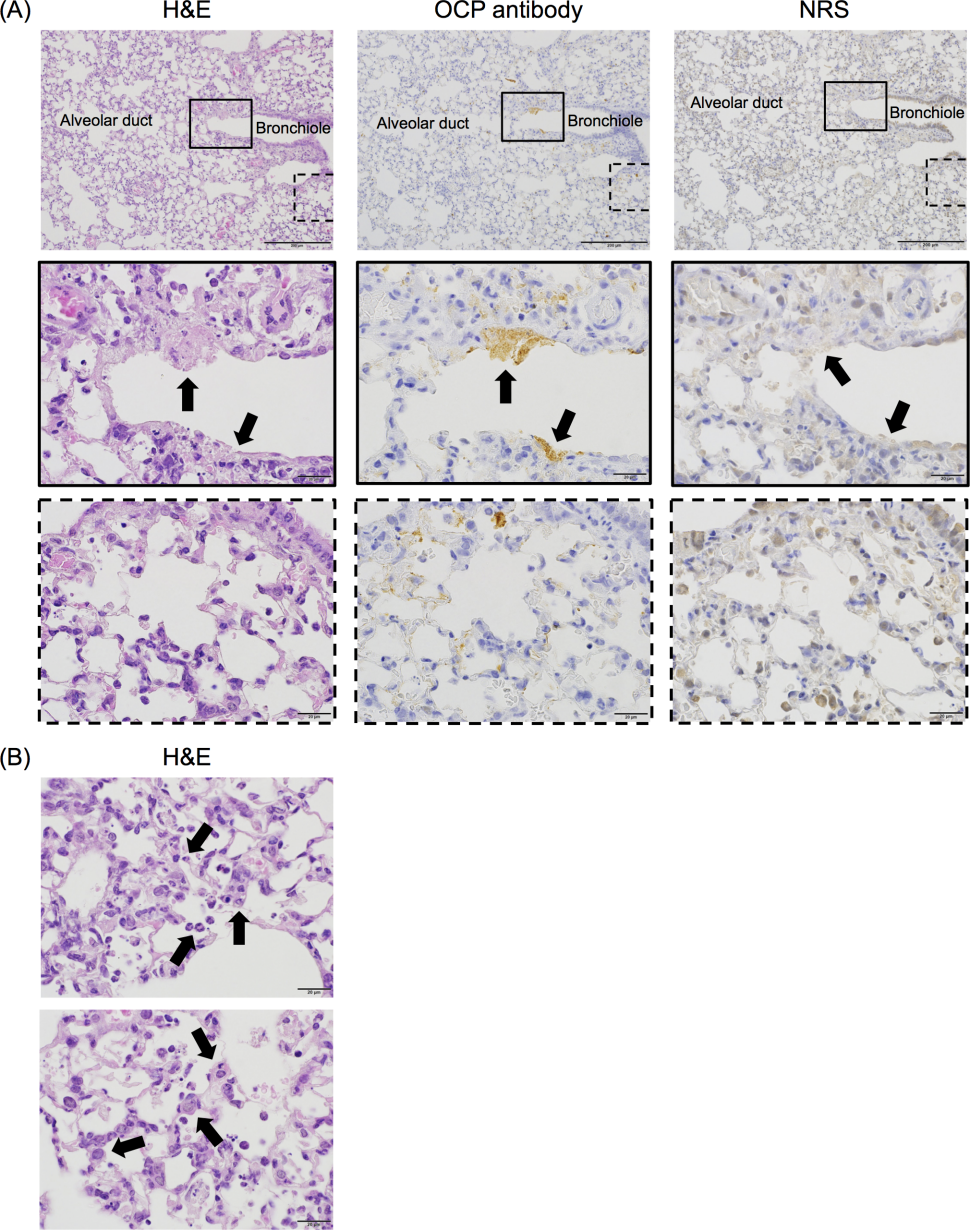
Histopathological findings of the lungs of BALB/c mice infected with PRV-MB. The lungs were obtained from PRV-MB-1.0×10^6^ PFU mice on the 4th DPI. H&E staining (A, left panels, and B) and IHC analysis with an OCP antibody (A, middle panels) or with NRS (A, right panels) were performed. The H&E staining and IHC analysis with an OCP antibody or with NRS of the lung at 200× magnification (A, upper panels), of a bronchiole at 1000× magnification (A, middle panels), and of an alveolus at 1000× magnification (A, lower panels) are shown. The black boxes in the upper panels indicate the necrotic lesion, which was positive for PRV-MB antigen (A, upper-middle panel). These areas are shown at higher magnification in the middle panels. The black arrows in the middle panels indicate the area of bronchiolar epithelial cell necrosis. The black dotted boxes in the upper panels indicate the PRV-MB antigen-positive lesion in the alveolar area and the images are enlarged in the lower panels (A, lower-middle panel). The PRV-MB antigen-positive lesion did not show a positive reaction in the IHC analysis, in which NRS was used instead of the OCP antibody (A, right panels). The scale bars in A (upper panels) indicate 200 μm, whereas those in A (middle panels), and A (lower panels) indicate 20 μm. H&E staining images of the alveoli at 1000× magnification (B) are shown. The black arrows in the upper and lower panels indicate neutrophils and type II pneumocytes, respectively. The scale bars in (B) indicate 20 μm.

A pathological examination revealed tissue damage and inflammation (i.e., necrosis and the accumulation of inflammatory cells including lymphocytes) in the lower respiratory tract, including the bronchiole and alveoli, in which viral antigens were detected in IHC analysis by using the OCP antibody, on the 4th DPI (Fig 3A, left and middle panels). Neutrophils and type II pneumocytes infiltrated to the alveoli and alveolar walls, and tissue damage in the lungs was detected (Fig 3B). The PRV antigen-positive lesions revealed in the IHC analysis by using the OCP antibody showed negative reaction in the IHC analysis using NRS (Fig 3A, right panels). No pathological changes or viral antigens were detected in the other tissues examined.

### Viral genome loads in the lungs according to the time course

**Fig 4:**
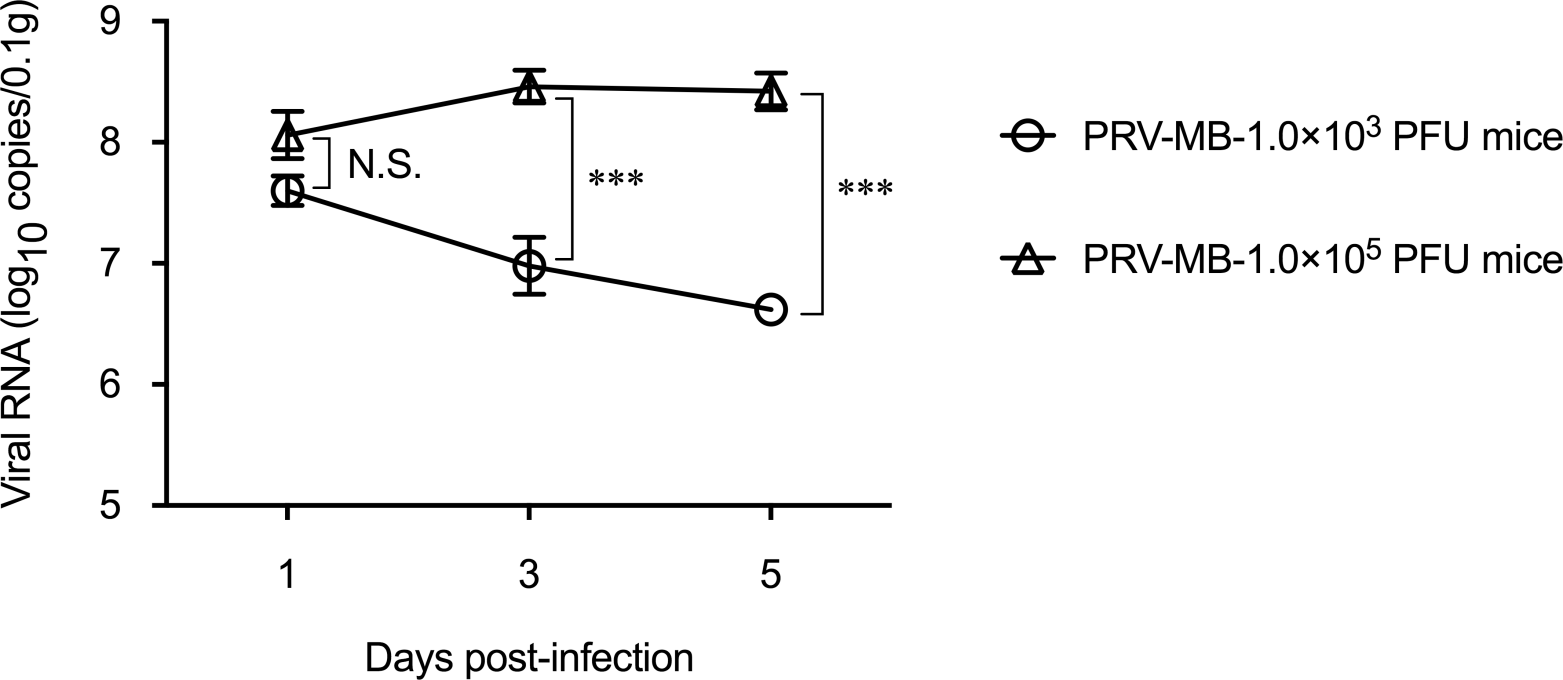
Temporal changes in the viral RNA loads in the lungs of the BALB/c mice infected with PRV-MB. The lungs were obtained from PRV-MB-1.0×10^3^ mice or PRV-MB-1.0×10^5^ PFU mice on the 1st, 3rd, and 5th DPI (5 mice per group for each day). Error bars indicate the standard error. N.S., not significant. ***, statistically significant (p < 0.0001).

The viral RNA in the lungs of the PRV-MB-1.0×10^3^ PFU mice or the PRV-MB-1.0×10^5^ PFU mice was determined throughout the course of infection (Fig 4). On the 1st DPI, the viral RNA load in the lungs of the PRV-MB-1.0×10^5^ PFU mice was similar to that of the PRV-MB-1.0×10^3^ PFU mice. In contrast, on the 3rd and 5th DPI, the viral RNA load in the lungs of the PRV-MB-1.0×10^5^ PFU mice was significantly higher in comparison to the PRV-MB-1.0×10^3^ PFU mice.

**Fig 5:**
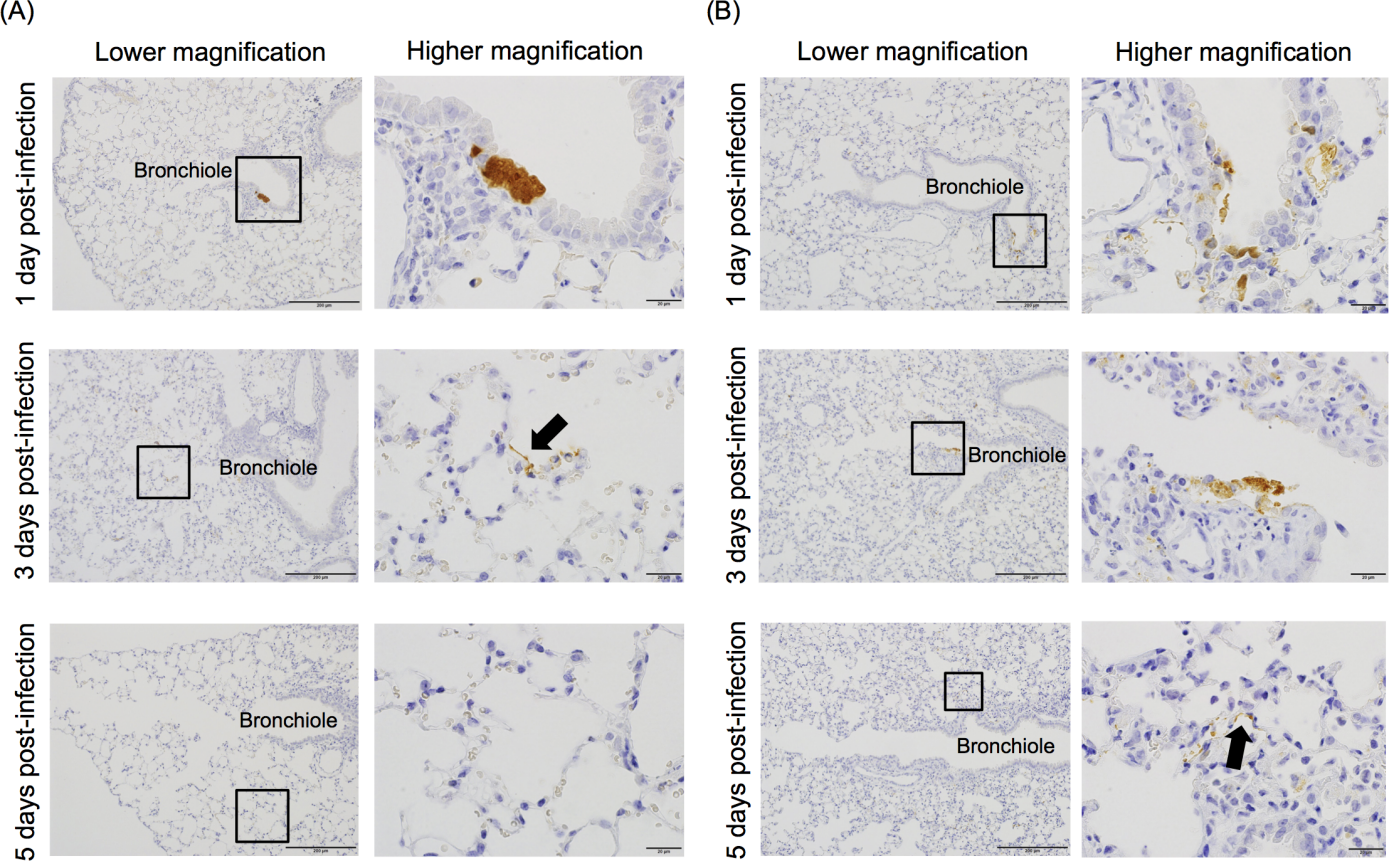
Histopathological findings in the lungs of BALB/c mice infected with PRV-MB on the 1st, 3rd, and 5th DPI. The lungs were obtained from the PRV-MB-1.0×10^3^ PFU mice (A) and the PRV-MB-1.0×10^5^ PFU mice (B) on the 1st, 3rd, and 5th DPI. IHC analysis of the lungs was performed using the OCP antibody for detection of PRV antigen. The right panels show enlarged views of the areas of interesting lesions (black squares in the left panels). The magnification levels of the left and right panels are 200× and 1000×, respectively. Black arrows indicate the pneumocytes that were positive for PRV-MB antigen. The scale bars in the left and right panels indicate 200 μm and 20 μm, respectively.

**Fig 6:**
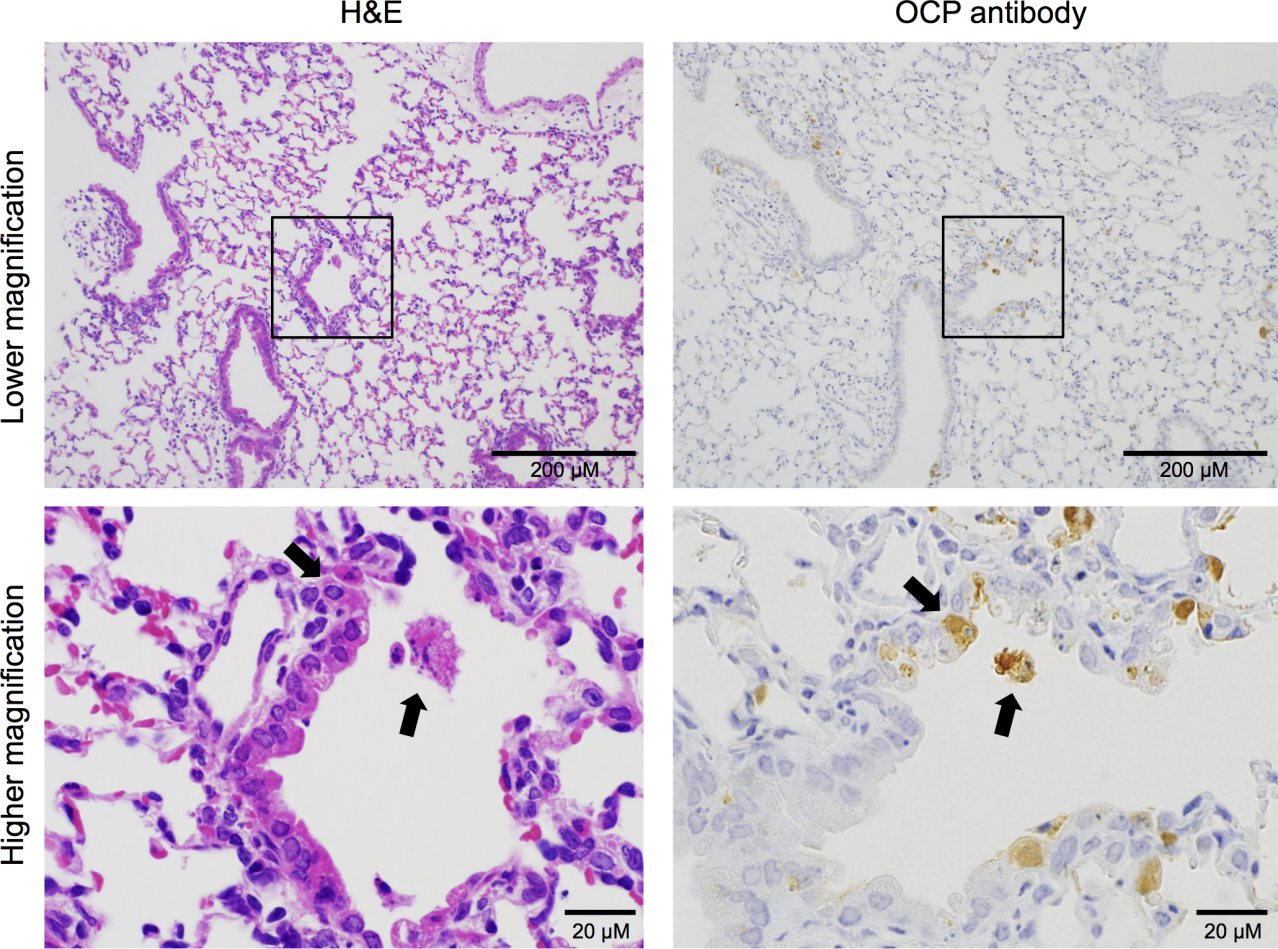
Histopathological findings in the terminal bronchiole of the BALB/c mice infected with a lethal dose of PRV-MB on the 1st DPI. The lungs from the PRV-MB-1.0×10^5^ PFU mice were obtained on the 1st DPI. The lungs were H&E stained (left panels) and tested immunohistochemically using the OCP antibody (right panels). The lower panels show enlarged views of areas of interesting lesions (black squares in the upper panels). The black arrows indicate necrotic cells and cellular debris. The magnification levels of the upper and lower panels are 200× and 1000×, respectively. The scale bars in the upper and lower panels indicate 200 μm and 20 μm, respectively.

The presence of viral antigens in the lungs of the PRV-MB-1.0×10^3^ PFU mice and the PRV-MB-1.0×10^5^ PFU mice was investigated immunohistochemically on the 1st, 3rd, and 5th DPI. Viral antigens were detected in the bronchial epithelium of the PRV-MB-1.0×10^5^ PFU mice on the 1st DPI (Fig 5B, upper panel), in the alveolar duct, alveoli, and bronchial epithelium on the 3rd DPI, and in the alveolar area on the 5th DPI (Fig 5B, middle and lower panels). Cellular damage characterized by positive nuclear aggregation, cellular atrophy, and cellular debris was detected in the terminal bronchioles, which was also positive for PRV-MB antigen (Fig 6). PRV-MB caused extensive and massive pulmonary infection in the PRV-MB-1.0×10^5^ PFU mice. In contrast, few viral antigens were detected in the bronchial epithelium of the PRV-MB-1.0×10^3^ PFU mice on the 1st and 3rd DPI (Fig 5A, upper and middle panels, respectively), and no viral antigens were detected in the bronchial epithelium or alveoli on the 5th DPI (Fig 5A, lower panel).

### Protection from lethal PRV-MB infection by induction of an immune response to PRV-MB in mice

**Fig 7:**
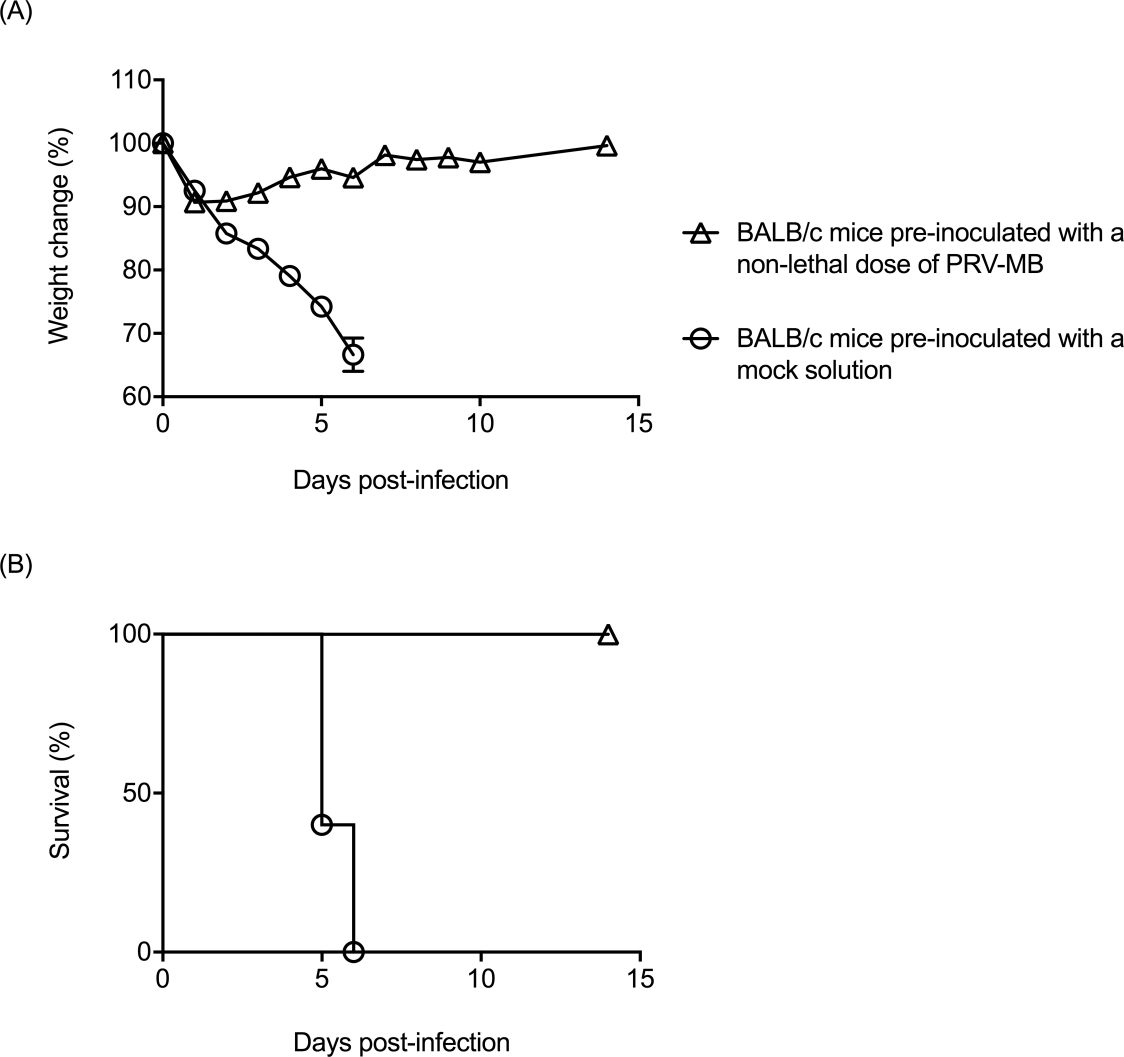
**Changes in the body weight (A) and survival rate (B) in BALB/c mice challenged with a lethal dose of PRV-MB after non-lethal PRV-MB infection.** BALB/c mice were pre-inoculated with a non-lethal dose (1.0 × 10^3^ PFU) of PRV-MB or with mock solution, followed by infection with 1.0 × 10^5^ PFU of PRV-MB on the 35th day after the first inoculation (5 mice per group). Error bars indicate the standard error.

The serum neutralizing antibody titers induced in the mice inoculated with 1.0 × 10^3^ PFU (non-lethal dose) of PRV-MB on the 27th DPI were between 640 and 2560. The mice were then challenged with 1.0 × 10^5^ PFU of PRV-MB on the 35th day after the first inoculation with a non-lethal dose of PRV-MB. All of these mice survived, whereas all of the control mice died by the 6th DPI (Fig 7).

### Effect of antiserum in the treatment of PRV-MB infection in mice

**Fig 8:**
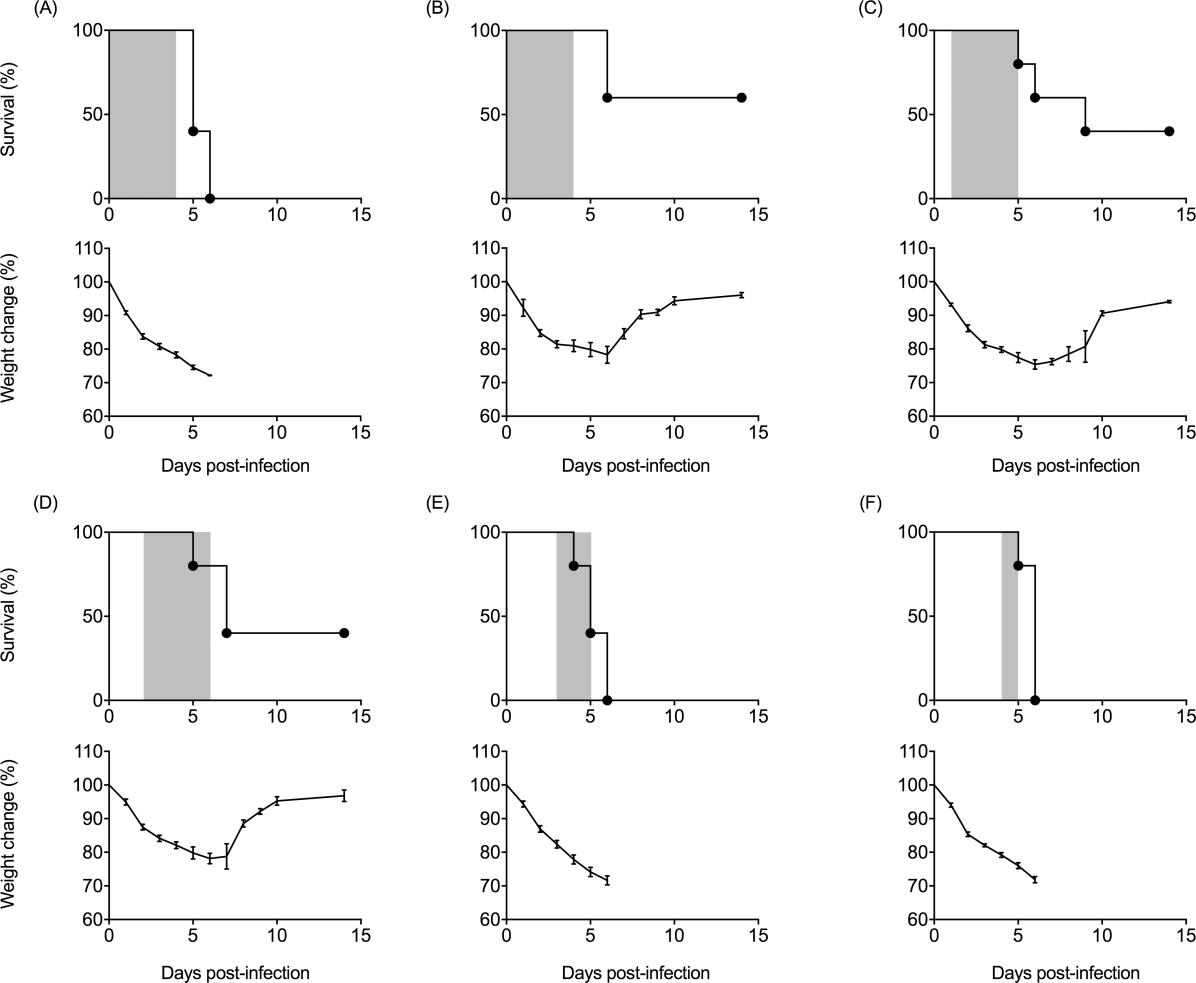
Changes in the survival rate (upper panels) and body weight (lower panels) in BALB/c mice treated with antiserum after lethal PRV-MB infection. The PRV-MB-1.0×10^5^ PFU mice were treated with the intraperitoneal administration of antiserum once daily during periods surrounded by the gray boxes (5 mice per group). Antiserum with a PRV-MB-specific serum neutralizing antibody titer of 1:10,240 was diluted 4-fold with PBS. The volume of the antiserum administered daily was 100 μL. The treatment was initiated at just after 1 h after the virus challenge (B) or from the 1st (C), 2nd (D), 3rd (E), and 4th (F) DPI. The control mice were administered the same volume of control serum, PRV antibody negative mouse serum diluted with PBS (A). Error bars indicate the standard error.

A mixture of the serum sample collected from mice infected with PRV-MB was used as an antiserum with a PRV-MB-specific serum neutralizing antibody titer of 10,240. The administration of antiserum to the mice that had been infected with 1.0 × 10^5^ PFU of PRV-MB showed a protective effect: the survival rate of the anti-serum-treated mice was 60%, whereas all of the control mice died (Fig 8). When the antiserum treatment was initiated on the 1st or 2nd DPI, taking the day on which the mice were infected with PRV-MB as day 0, 40% of the mice survived, whereas the control mice and the mice in which the treatment was initiated on the 3rd DPI or later died by the 6th DPI. The body weight reduction in these groups was similar to that of the control group.

### Virulence and pathogenicity of PRV-Samal-24 in BALB/c mice

Nine-week-old BALB/c mice were infected with a graded dosage (1.0 × 10^3^–1.0 × 10^6^ PFU) of PRV-Samal-24. The intranasal inoculation of the mice with PRV-Samal-24 led to fatal outcomes. The LD_50_ of PRV-Samal-24 for BALB/c mice was determined to be 4.2 × 10^3^ PFU/head.

**Fig 9:**
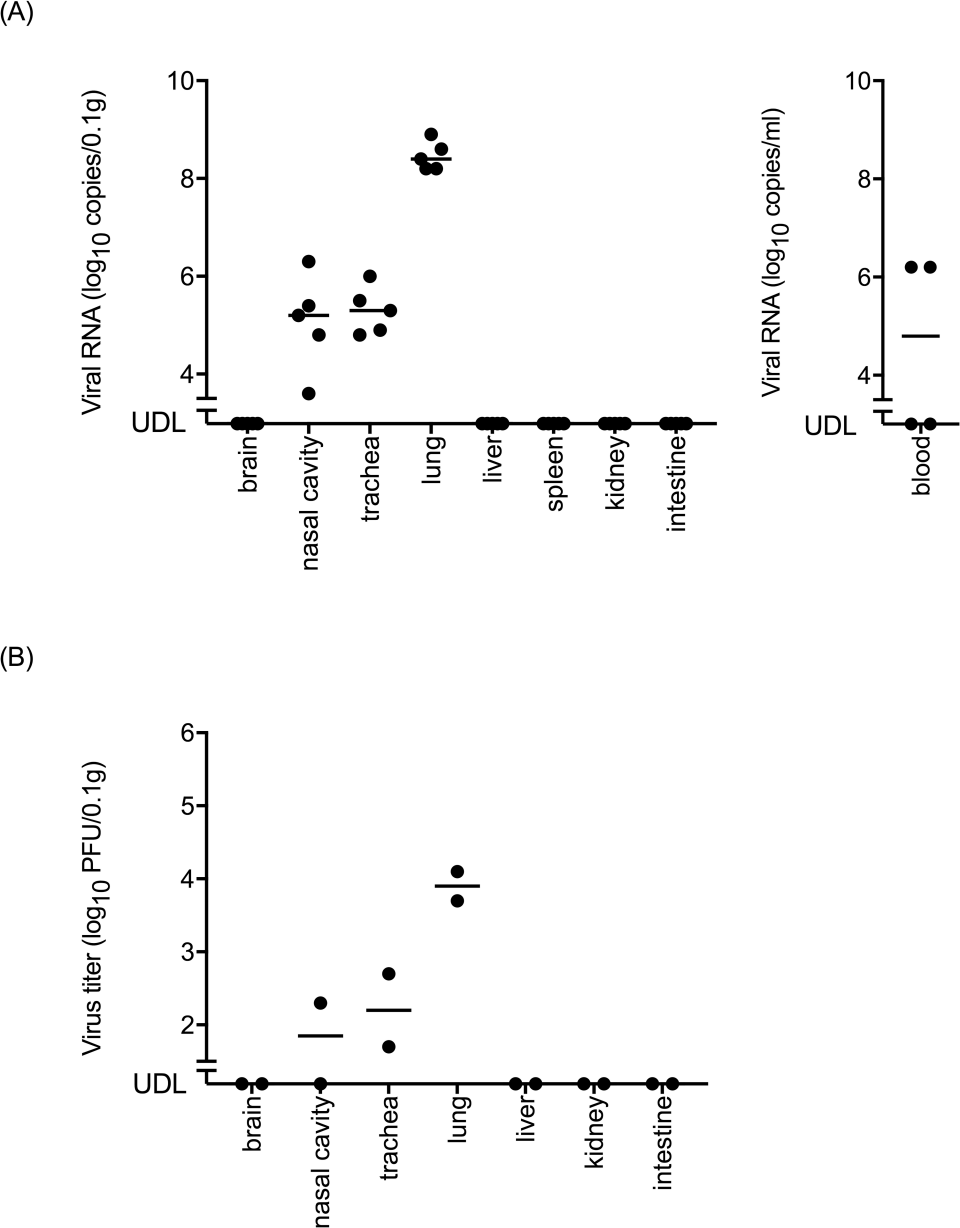
**Viral RNA loads in organs and the blood (A) and infectious PRV titers in the organs (B) of BALB/c mice infected with PRV-Samal-24.** (A) The organs and the blood samples were obtained from the PRV-Samal-24-1.0×10^5^ PFU mice on the 5th DPI (5 mice per group). Four PRV-Samal-24-1.0×10^5^ PFU mice were sacrificed on the 5th DPI due to their reaching the humane endpoint. Another mouse was found dead on the 5th DPI; therefore, a blood sample could not be obtained. Therefore, the viral RNA loads in each organ and those in the blood were determined for 5 and 4 mice, respectively. The left and right panels indicate the viral RNA in each organ and the blood of the PRV-Samal-24-1.0×10^5^ PFU mice, respectively. (B) The organs were obtained from the PRV-Samal-24-1.0×10^6^ PFU mice on the 4th DPI (2 mice per group). UDL, under detection limit.

Among the respiratory organs, viral RNA was detected in the lungs of the mice infected with PRV-Samal-24; the viral RNA copy numbers in the lungs were up to 3.7 × 10^8^ copies/0.1 g on average (Fig 9A, left panel). Viral RNA was also detected in the blood (maximum level, 1.8 × 10^6^ copies/ml) (Fig 9A, right panel). In addition, the infectious virus was isolated from the respiratory tract organs (Fig 9B). The infectious dose of PRV-Samal-24 was the highest in the lungs among the tissues tested with the dose being up to 9.5 × 10^3^ PFU/0.1 g on average.

Pathological examination of the PRV-Samal-24-1.0×10^6^ PFU mice revealed inflammatory lesions in the lungs (by H&E staining), and viral antigens were also detected, especially from the bronchioles to the alveoli (by IHC staining) as was observed in the mice infected with PRV-MB (S2 Fig).

## Discussion

The present study showed through virological and pathological examinations that the lung was the principle target organ of PRV replication after intranasal inoculation in BALB/c mice. PRV mainly replicated in the bronchiolar epithelium by the 3rd DPI. The bronchiolar epithelium is composed of ciliated and nonciliated cells, such as clara cells and goblet cells, which are classified as secretory cells [22]. Morphologically, the PRV antigen-positive cells were likely to be clara cells and goblet cells. PRV infection caused severe inflammation in the lungs of the mice on the 4th DPI (acute phase). Morphologically, PRV mainly replicated in the pneumocyte-like cells, and the PRV antigen-positive cells were likely to be type I pneumocytes, which are involved in the process of gas exchange between the alveoli and blood [23]. Mammalian orthoreovirus, which is classified to the genus *Orthoreovirus* in the family *Reoviridae*, was reported to replicate in type I pneumocytes and was shown to induce severe pneumonia in some rodent species, including mice and rats [24, 25]. Type I pneumocytes might be a critical replication site for PRV. It was assumed that fatal outcomes were induced in mice infected with a lethal dose of PRV due to a decrease in respiratory function that occurred as a result of the destruction of the bronchiolar epithelial cells and pneumocytes. In this study, the cell types, in which PRV replicated, were identified only by morphological observation. Further studies are needed to elucidate the primary target cells, which are infected with PRV and in which PRV replicates. The high-titer PRV genome and infectious PRV were detected in the lungs of the mice infected with a lethal dose of PRV on the 4th to 6th DPI (acute phase) (Figs 2 and 9). BALB/c mice were susceptible to PRV and developed RTIs, similarly to humans. Demonstration of infectious PRV in lungs indicates that PRV definitely replicated there (Figs 2B and 9B). Koch’s postulates (i.e., isolation of PRV from patients with RTIs, induction of RTI in mice by infection with PRV, and detection of infectious PRV in respiratory organs of mice infected with PRV) support a causal role of PRV infection in the development of respiratory tract diseases in humans [26]. Although all of the cases of PRV infection in humans showed symptoms associated with RTI, it is evident that the clinical characteristics of PRV infections in humans have not been fully elucidated. It is possible that PRV causes more severe infections than have previously been reported.

We evaluated the utility of the newly developed mouse model of PRV infection. Immunity to PRV was induced by non-lethal infection, and it protected the mice from lethal infection with PRV (Fig 7). The early initiation of antiserum treatment was effective in the treatment of lethal PRV infection (Fig 8). These results suggest that BALB/c mice may serve as a useful animal model for evaluating the efficacy of vaccines and therapeutic agents for PRV.

The pathogenicity of PRV-Samal-24 was evaluated in this mouse model. Similarly to PRV-MB, PRV-Samal-24 caused viremia and respiratory disease in the BALB/c mice. The amino acid identities (encoded by each gene segment) between PRV-Samal-24 and PRV-MB were as follows: cell attachment protein region of the S1 segment, 82%; p10 region of the S1 segment, 100%; p17 region of the S1 segment, 94%; inner-capsid protein region of the S2 segment, 97%; sigma NS region of the S3 segment, 97%; OCP region of the S4 segment, 97%; minor inner-capsid protein region of the M1 segment, 94%; major outer-capsid protein region of the M2 segment, 95%, mu NS region of the M3 segment, 91%, guanylyltransferase region of the L1 segment, 94%, RNA polymerase region of the L2 segment, 98%, and major inner-capsid protein region of the L3 segment, 98%. PRV-Samal-24 and PRV-MB were also reported to show cross-reactivity in an immunofluorescence assay [15]. As both the *in vivo* and *in vitro* characteristics of PRV-Samal-24 are similar to PRV-MB, it is possible for bat-borne PRV to cause illness in humans.

In conclusion, a BALB/c mouse model of PRV infection, in which PRV caused acute RTI, was developed. Immunocompetent BALB/c mice were sensitive to PRV, when the mice were infected with PRV intranasally. This model might be useful for analyzing the pathogenicity of PRV in mice and for evaluating the efficacy of vaccines and therapeutic agents that will be developed to prevent and treat PRV infection. This model is also useful for further studies on PRV infections in vivo.

## Supporting information

S1 Fig Validation of the specificity of the OCP antibody in the detection of PRV antigen in IHC. The SARS-mouse-lung (left panels) and mock-mouse-lung (right panels) [21] were examined by IHC with using the OCP antibody. The IHC signals in the lung at 100× magnification (upper panels), the bronchiole at 200× magnification (middle panels), and the alveolus at 1000× magnification (lower panels) are shown. No signal, which indicates non-specific reaction of the OCP antibody, was detected in the SARS-mouse-lung, in which severe inflammation was found on H&E staining [21], and mock-mouse-tissue. The scale bars in upper and middle panels indicate 200 μm, whereas those in lower panels indicate 20 μm.
(TIF)

S2 Fig Histopathological findings of the lungs of BALB/c mice infected with PRV-Samal-24. The lungs were obtained from PRV-Samal-24-1.0×10^6^ PFU mice on the 4th DPI. H&E staining (left panels) and IHC with an OCP antibody (right panels) were performed. The H&E staining and IHC with an OCP antibody of the lung at 100× magnification (upper panels) and of a bronchiole and an alveolus at 400× magnification (lower panels) are shown. The black boxes in the upper panels were shown at higher magnification in the lower panels. The black arrows in the lower panels indicate the bronchiolar epithelial cell necrosis, which was positive for PRV-Samal-24 antigen. The red arrows in the lower-right panel indicate the PRV-Samal-24 antigen-positive pneumocytes. The scale bars in the upper panels indicate 500 μm, whereas those in the lower panels indicate 100 μm.
(TIF)

## References

[1] Gard GP, Marshall ID. Nelson Bay virus. A novel reovirus. Archiv fur Die Gesamte Virusforschung. 1973;43(1):34–42. Epub 1973/01/01. . 4367379 10.1007/BF01249346

[2] Chua KB, Crameri G, Hyatt A, Yu M, Tompang MR, Rosli J, et al A previously unknown reovirus of bat origin is associated with an acute respiratory disease in humans. Proceedings of the National Academy of Sciences of the United States of America. 2007;104(27):11424–9. Epub 2007/06/27. doi: 10.1073/pnas.0701372104 ; PubMed Central PMCID: PMCPMC1899191. 17592121 10.1073/pnas.0701372104 PMC1899191

[3] Chua KB, Voon K, Crameri G, Tan HS, Rosli J, McEachern JA, et al Identification and characterization of a new orthoreovirus from patients with acute respiratory infections. PloS One. 2008;3(11):e3803 Epub 2008/11/26. doi: 10.1371/journal.pone.0003803 ; PubMed Central PMCID: PMCPMC2583042. 19030226 10.1371/journal.pone.0003803 PMC2583042

[4] Cheng P, Lau CS, Lai A, Ho E, Leung P, Chan F, et al A novel reovirus isolated from a patient with acute respiratory disease. Journal of Clinical Virology. 2009;45(1):79–80. Epub 2009/04/10. doi: 10.1016/j.jcv.2009.03.001 . 19356975 10.1016/j.jcv.2009.03.001

[5] Chua KB, Voon K, Yu M, Keniscope C, Abdul Rasid K, Wang LF. Investigation of a potential zoonotic transmission of orthoreovirus associated with acute influenza-like illness in an adult patient. PloS One. 2011;6(10):e25434 Epub 2011/10/25. doi: 10.1371/journal.pone.0025434 ; PubMed Central PMCID: PMCPMC3192755. 22022394 10.1371/journal.pone.0025434 PMC3192755

[6] Wong AH, Cheng PK, Lai MY, Leung PC, Wong KK, Lee WY, et al Virulence potential of fusogenic orthoreoviruses. Emerging Infectious Diseases. 2012;18(6):944–8. Epub 2012/05/23. doi: 10.3201/eid1806.111688 ; PubMed Central PMCID: PMCPMC3358160. 22608100 10.3201/eid1806.111688 PMC3358160

[7] Yamanaka A, Iwakiri A, Yoshikawa T, Sakai K, Singh H, Himeji D, et al Imported case of acute respiratory tract infection associated with a member of species Nelson Bay orthoreovirus. PloS One. 2014;9(3):e92777 Epub 2014/03/29. doi: 10.1371/journal.pone.0092777 ; PubMed Central PMCID: PMCPMC3965453. 24667794 10.1371/journal.pone.0092777 PMC3965453

[8] Tan YF, Teng CL, Chua KB, Voon K. Pteropine orthoreovirus: An important emerging virus causing infectious disease in the tropics? Journal of Infection in Developing Countries. 2017;11(3):215–9. Epub 2017/04/04. doi: 10.3855/jidc.9112 . 28368854 10.3855/jidc.9112

[9] Singh H, Shimojima M, Ngoc TC, Quoc Huy NV, Chuong TX, Le Van A, et al Serological evidence of human infection with Pteropine orthoreovirus in Central Vietnam. Journal of Medical Virology. 2015;87(12):2145–8. Epub 2015/05/27. doi: 10.1002/jmv.24274 . 26010233 10.1002/jmv.24274 PMC5157728

[10] Voon K, Tan YF, Leong PP, Teng CL, Gunnasekaran R, Ujang K, et al Pteropine orthoreovirus infection among out-patients with acute upper respiratory tract infection in Malaysia. Journal of Medical Virology. 2015;87(12):2149–53. Epub 2015/06/25. doi: 10.1002/jmv.24304 . 26106066 10.1002/jmv.24304 PMC7167058

[11] Pritchard LI, Chua KB, Cummins D, Hyatt A, Crameri G, Eaton BT, et al Pulau virus; a new member of the Nelson Bay orthoreovirus species isolated from fruit bats in Malaysia. Archives of Virology. 2006;151(2):229–39. Epub 2005/10/06. doi: 10.1007/s00705-005-0644-4 . 16205863 10.1007/s00705-005-0644-4

[12] Du L, Lu Z, Fan Y, Meng K, Jiang Y, Zhu Y, et al Xi River virus, a new bat reovirus isolated in southern China. Archives of Virology. 2010;155(8):1295–9. Epub 2010/05/25. doi: 10.1007/s00705-010-0690-4 . 20495835 10.1007/s00705-010-0690-4 PMC7087202

[13] Hu T, Qiu W, He B, Zhang Y, Yu J, Liang X, et al Characterization of a novel orthoreovirus isolated from fruit bat, China. BMC Microbiology. 2014;14:293 Epub 2014/12/01. doi: 10.1186/s12866-014-0293-4 ; PubMed Central PMCID: PMCPMC4264558. 25433675 10.1186/s12866-014-0293-4 PMC4264558

[14] Lorusso A, Teodori L, Leone A, Marcacci M, Mangone I, Orsini M, et al A new member of the Pteropine orthoreovirus species isolated from fruit bats imported to Italy. Infection, Genetics and Evolution. 2015;30:55–8. Epub 2014/12/17. doi: 10.1016/j.meegid.2014.12.006 . 25497353 10.1016/j.meegid.2014.12.006

[15] Taniguchi S, Maeda K, Horimoto T, Masangkay JS, Puentespina R Jr., Alvarez J, et al First isolation and characterization of pteropine orthoreoviruses in fruit bats in the Philippines. Archives of Virology. 2017 Epub 2017/02/13. doi: 10.1007/s00705-017-3251-2 . 28190201 10.1007/s00705-017-3251-2

[16] Kawagishi T, Kanai Y, Tani H, Shimojima M, Saijo M, Matsuura Y, et al Reverse genetics for fusogenic bat-borne orthoreovirus associated with acute respiratory tract infections in humans: role of outer capsid protein sigmaC in viral replication and pathogenesis. PLoS Pathogens. 2016;12(2):e1005455 Epub 2016/02/24. doi: 10.1371/journal.ppat.1005455 ; PubMed Central PMCID: PMCPMC4762779. 26901882 10.1371/journal.ppat.1005455 PMC4762779

[17] Singh H, Yoshikawa T, Kobayashi T, Fukushi S, Tani H, Taniguchi S, et al Rapid whole genome sequencing of Miyazaki-Bali/2007 Pteropine orthoreovirus by modified rolling circular amplification with adaptor ligation—next generation sequencing. Scientific Reports. 2015;5:16517 Epub 2015/11/13. doi: 10.1038/srep16517 ; PubMed Central PMCID: PMCPMC4642344. 26558341 10.1038/srep16517 PMC4642344

[18] Ramakrishnan MA. Determination of 50% endpoint titer using a simple formula. World Journal of Virology. 2016;5(2):85–6. Epub 2016/05/14. doi: 10.5501/wjv.v5.i2.85 ; PubMed Central PMCID: PMCPMC4861875. 27175354 10.5501/wjv.v5.i2.85 PMC4861875

[19] Kotani O, Naeem A, Suzuki T, Iwata-Yoshikawa N, Sato Y, Nakajima N, et al Neuropathogenicity of two Saffold virus type 3 isolates in mouse models. PloS One. 2016;11(2):e0148184 Epub 2016/02/02. doi: 10.1371/journal.pone.0148184 ; PubMed Central PMCID: PMCPMC4734772. 26828718 10.1371/journal.pone.0148184 PMC4734772

[20] Singh H, Shimojima M, Fukushi S, Fukuma A, Tani H, Yoshikawa T, et al Serologic assays for the detection and strain identification of Pteropine orthoreovirus. Emerging Microbes & Infections. 2016;5:e44 Epub 2016/05/12. doi: 10.1038/emi.2016.35 ; PubMed Central PMCID: PMCPMC4893542. 27165561 10.1038/emi.2016.35 PMC4893542

[21] Nagata N, Iwata N, Hasegawa H, Fukushi S, Harashima A, Sato Y, et al Mouse-passaged severe acute respiratory syndrome-associated coronavirus leads to lethal pulmonary edema and diffuse alveolar damage in adult but not young mice. The American Journal of Pathology. 2008;172(6):1625–37. Epub 2008/05/10. doi: 10.2353/ajpath.2008.071060 ; PubMed Central PMCID: PMCPMC2408422. 18467696 10.2353/ajpath.2008.071060 PMC2408422

[22] Plopper CG, Mariassy AT, Wilson DW, Alley JL, Nishio SJ, Nettesheim P. Comparison of nonciliated tracheal epithelial cells in six mammalian species: ultrastructure and population densities. Experimental Lung Research. 1983;5(4):281–94. Epub 1983/12/01. . 6662075 10.3109/01902148309061521

[23] Herzog EL, Brody AR, Colby TV, Mason R, Williams MC. Knowns and unknowns of the alveolus. Proceedings of the American Thoracic Society. 2008;5(7):778–82. Epub 2008/09/02. doi: 10.1513/pats.200803-028HR ; PubMed Central PMCID: PMCPMC2645265. 18757317 10.1513/pats.200803-028HR PMC2645265

[24] Gauvin L, Bennett S, Liu H, Hakimi M, Schlossmacher M, Majithia J, et al Respiratory infection of mice with mammalian reoviruses causes systemic infection with age and strain dependent pneumonia and encephalitis. Virology Journal. 2013;10:67 Epub 2013/03/05. doi: 10.1186/1743-422X-10-67 ; PubMed Central PMCID: PMCPMC3605257. 23453057 10.1186/1743-422X-10-67 PMC3605257

[25] Morin MJ, Warner A, Fields BN. Reovirus infection in rat lungs as a model to study the pathogenesis of viral pneumonia. Journal of Virology. 1996;70(1):541–8. Epub 1996/01/01. ; PubMed Central PMCID: PMCPMC189842. 8523567 10.1128/jvi.70.1.541-548.1996 PMC189842

[26] Kaufmann SH, Schaible UE. 100th anniversary of Robert Koch's Nobel prize for the discovery of the tubercle bacillus. Trends in Microbiology. 2005;13(10):469–75. Epub 2005/08/23. doi: 10.1016/j.tim.2005.08.003 . 16112578 10.1016/j.tim.2005.08.003

