# Supplementary figures and images for: Virulence, pathology, and pathogenesis of Pteropine orthoreovirus (PRV) in BALB/c mice: Development of an animal infection model for PRV
Source: PLoS Negl Trop Dis. 2017 Dec 14;11(12):e0006076. doi: 10.1371/journal.pntd.0006076 (PMC5730109; doi:10.1371/journal.pntd.0006076)

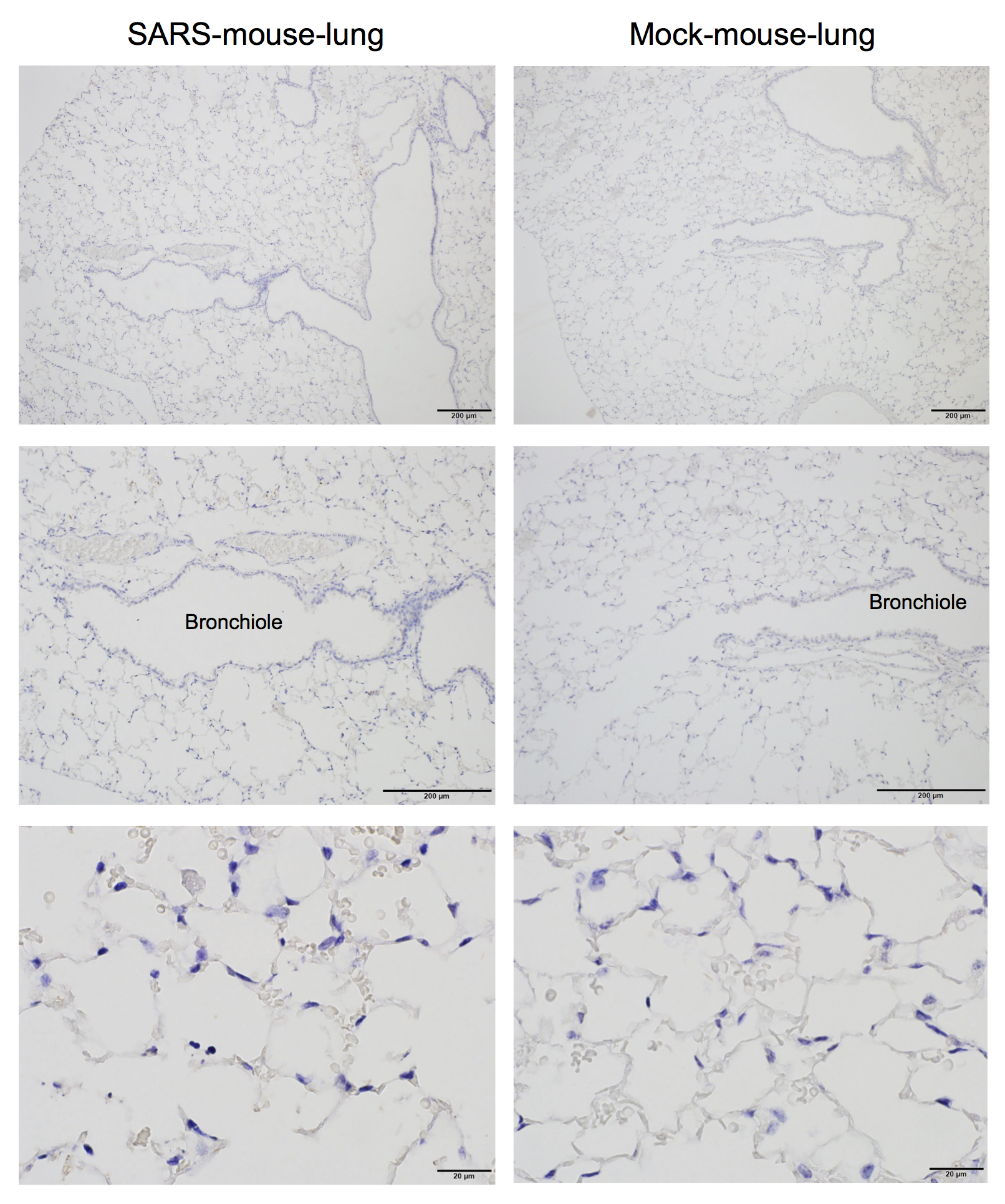

Supplement: S1 Fig — The SARS-mouse-lung (left panels) and mock-mouse-lung (right panels) [21] were examined by IHC with using the OCP antibody. The IHC signals in the lung at 100× magnification (upper panels), the bronchiole at 200× magnification (middle panels), and the alveolus at 1000× magnification (lower panels) are shown. No signal, which indicates non-specific reaction of the OCP antibody, was detected in the SARS-mouse-lung, in which severe inflammation was found on H&E staining [21], and mock-mouse-tissue. The scale bars in upper and middle panels indicate 200 μm, whereas those in lower panels indicate 20 μm. (TIF) [file pntd.0006076.s001.tif]

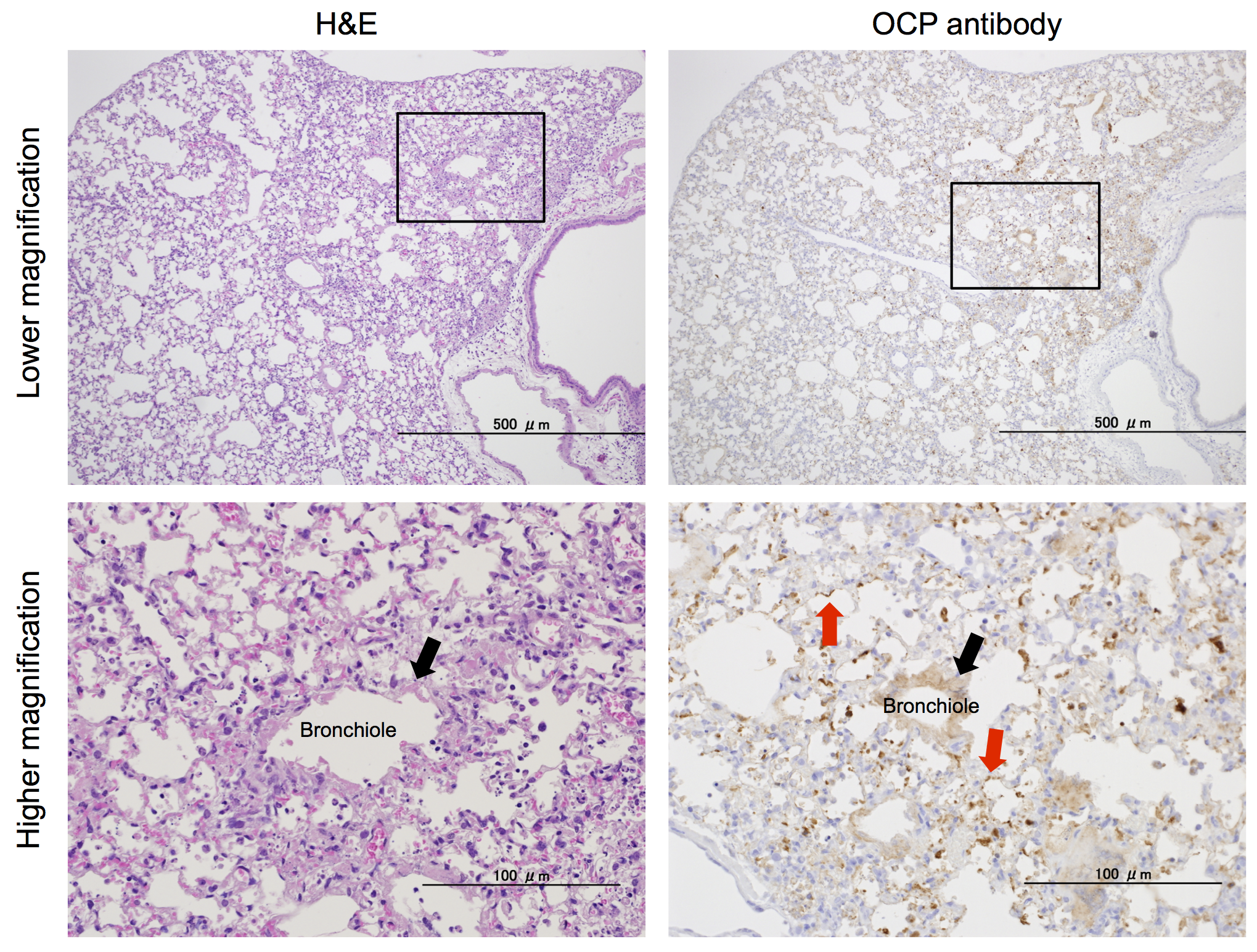

Supplement: S2 Fig — The lungs were obtained from PRV-Samal-24-1.0×10^6^ PFU mice on the 4th DPI. H&E staining (left panels) and IHC with an OCP antibody (right panels) were performed. The H&E staining and IHC with an OCP antibody of the lung at 100× magnification (upper panels) and of a bronchiole and an alveolus at 400× magnification (lower panels) are shown. The black boxes in the upper panels were shown at higher magnification in the lower panels. The black arrows in the lower panels indicate the bronchiolar epithelial cell necrosis, which was positive for PRV-Samal-24 antigen. The red arrows in the lower-right panel indicate the PRV-Samal-24 antigen-positive pneumocytes. The scale bars in the upper panels indicate 500 μm, whereas those in the lower panels indicate 100 μm. (TIF) [file pntd.0006076.s002.tif]
